# The growing importance of lesion volume as a prognostic factor in patients with multiple brain metastases treated with stereotactic radiosurgery

**DOI:** 10.1002/cam4.1352

**Published:** 2018-02-14

**Authors:** David M. Routman, Shelly X. Bian, Kevin Diao, Jonathan L. Liu, Cheng Yu, Jason Ye, Gabriel Zada, Eric L. Chang

**Affiliations:** ^1^ Department of Radiation Oncology Mayo Clinic Rochester Minnesota; ^2^ Department of Radiation Oncology Keck School of Medicine of USC Los Angeles California; ^3^ Harvard Medical School Boston Massachusetts; ^4^ Department of Radiology Washington University St. Louis Missouri; ^5^ Department of Neurological Surgery Keck School of Medicine of USC Los Angeles California

**Keywords:** Brain metastases, lesion number, stereotactic radiosurgery, tumor volume, whole‐brain radiation therapy

## Abstract

Stereotactic Radiosurgery (SRS) is considered standard of care for patients with 1–3 brain metastases (BM). Recent observational studies have shown equivalent OS in patients with 5+ BM compared to those with 2–4, suggesting SRS alone may be appropriate in these patients. We aim to review outcomes of patients treated with SRS with 2–4 versus 5+ BM. This analysis included consecutive patients from 1994 to 2015 treated with SRS. Of 1017 patients, we excluded patients with a single BM and patients without adequate survival data, resulting in 391 patients. All risk factors were entered into univariate analysis using Cox proportional hazards model, and significant factors were entered into multivariate analysis (MVA). We additionally analyzed outcomes after excluding patients with prior surgery or whole‐brain radiotherapy (WBRT). Median follow‐up was 7.1 months. Median KPS was 90, mean age was 59, and most common histologies were melanoma and lung. Median tumor volume was 3.41 cc. Patients with 2–4 BM had a median OS of 8.1 months compared to 6.2 months for those with 5+ BM (*P* = 0.0136). On MVA, tumor volume, KPS, and histology remained significant for OS, whereas lesion number did not. Similar results were found when excluding patients with prior surgery or WBRT. Rather than lesion number, the strongest prognostic factors for patients undergoing SRS were tumor volume >10 cc, KPS, and histology. BM number may therefore not be the most important criterion for candidacy for SRS. Patients with 5 or more BM should be considered for SRS.

## Introduction

Brain metastases (BM) develop in up to 20–40% of patients diagnosed with cancer, and the incidence is increasing as cancer patients are living longer with improving systemic therapy 1, 2. Radiation therapy (RT) is an integral component of the treatment of BM to improve local control, and in certain instances, overall survival (OS). Whole‐brain radiation therapy (WBRT) has traditionally been the standard for patients with multiple BM. However, there has been a trend toward increased use of stereotactic radiosurgery (SRS) for management of patients with single or <4 BM 3, 4.

The efficacy and toxicity of WBRT in comparison with and in addition to SRS have been evaluated in a randomized fashion and in a meta‐analysis 5. Recent trials have shown the addition of WBRT to SRS does not improve survival outcomes, and WBRT causes significant declines in neurocognition and overall quality of life (QOL) for patients with 1–3 BM 6, 7. For example, Alliance trial N0574 randomized patients with 1–3 BM to SRS versus SRS with WBRT and found no statistically significant difference in median OS, 10.4 months versus 7.4 months, respectively (*P* = 0.92). While WBRT after SRS improved local and regional control, it had no impact on OS and was associated with decreased cognitive function and decreased quality of life 3 months after treatment 7.

Furthermore, SRS can be performed in a single fraction which is more convenient for patients. Often, patients will require an interruption in systemic therapy while undergoing WBRT due to concern regarding increased toxicity with concurrent treatment. Thus, patients treated with SRS may end up ultimately receiving more systemic therapy in comparison with those undergoing WBRT 8. This is an especially important consideration in the age of newer targeted agents that can significantly impact survival and disease burden.

Stereotactic radiosurgery alone or in combination with other modalities is therefore generally accepted as the standard of care for patients with 1–3 BM. However, debate and uncertainty regarding the optimal management of patients with 4–5 or more BM remains. Yamamoto et al. have suggested equivalent outcomes for patients with four or more BM treated with SRS in comparison with patients with fewer BM 9, 10. There are no published randomized trials for SRS versus WBRT for patients with five or more BM.

In the absence of level 1 evidence addressing the optimal management of patients presenting with multiple BM, the aim of this study was to compare OS and identify prognostic factors in patients with 2–4 versus 5 or more BM. This analysis further elucidates considerations for the use of SRS in patients with more than 4 BM by reviewing patients treated at a single institution over the last two decades.

## Methods

### Study population

Institutional review board approval was obtained and retrospective review was performed of all consecutive patients with BM treated with SRS at our institution from 1994 to 2015. We included patients with two or more treated BM, and excluded patients who were missing critical baseline, treatment, or survival information. We additionally analyzed outcomes after excluding patients who were treated with prior neurosurgical resection or WBRT. We obtained patient and treatment information from electronic medical records and survival data from the institutional cancer registry.

### Radiation delivery

All patients were treated with single‐fraction Gamma Knife radiosurgery. Gamma Knife (Elekta Instruments Inc.) Model U was used from 1994 to 2000, Model C was used from 2000 to 2008, and Perfexion was used from 2008 to 2015. All patients were immobilized with a stereotactic head frame. The frame application was performed by a neurosurgeon utilizing four pins affixed to the cranium after the patient was provided conscious sedation by a member of the anesthesia team. Contrast‐enhanced thin slice magnetic resonance imaging (MRI) of the brain was then performed for target delineation and treatment planning.

Dose prescription was at the discretion of the treating team and in general accordance with RTOG 90‐05. For tumor diameter <2 cm, prescription was 20–24 Gy typically to 50% isodoline line. For tumors >2 cm and <3 cm diameter, dose was generally 18 Gy to 50% isodose line. For tumors >3 cm, we typically prescribe 15 Gy to 50% isodoseline. This prescription guideline is not modified for various primary cancers including nonsmall cell lung cancer, breast cancer, melanoma, renal cell cancer, and colorectal cancer. Tumor equivalent volumes are converted using the formula for volume of a sphere, (4/3) * π * *r* ^3^. A 2‐cm‐diameter cutoff corresponds to 4.19 cc volumetric cutoff. A 3‐cm‐diameter cutoff corresponds to a 14.1 cc volumetric cutoff. The decision to leave BM untreated at the time of SRS was per the radiation oncologist and neurosurgeon at the time of treatment, with generally small and asymptomatic BM as lesions less likely to be treated 11.

### Statistical analysis

The primary endpoint was overall survival (OS) in months. Time was calculated from the date of initial SRS treatment to the date of death. Censoring occurred at the date the patient was last known to be alive. All risk factors were defined at the time of initial SRS. Analyzed risk factors for survival included age, sex, tumor histology, performance status, graded prognostic assessment (GPA) score, recursive partitioning analysis (RPA) class, synchronous or metachronous diagnosis of BM, increase in number of BM from initial consultation to treatment, untreated BM, infratentorial BM, number of BM treated, total tumor volume (continuous and categorical <5 cc, 5–10 cc, >10 cc), and SRS dose.

Performance status was graded with the Karnofsky Performance Score (KPS) on a scale of 0 to 100. GPA was scored from 0 to 4 based on age, KPS, number of BM, and the presence of extracranial metastases 12. RPA was scored from 1 to 3 based on age, KPS, control of primary disease, and the presence of extracranial metastases 13. Synchronous diagnosis of BM was defined as discovery of BM within 3 months of the diagnosis of the primary cancer. Progression of BM was defined as an increase in number of identified BM between the initial diagnostic MRI and the MRI on the day of SRS treatment. Patients were defined as having untreated BM if not all identified BM were targeted with SRS. The total tumor volume is the total volume of BM treated with SRS as measured on MRI using Leksell GammaPlan (Elekta Inc. Stockholm, Sweden) software.

Baseline variables were compared using the Wilcoxon rank sum test, Pearson's chi‐squared test, and Fisher's exact test. OS was analyzed using the Kaplan–Meier method with significance testing with the log rank test. We performed two analyses; Analysis 1 included all patients. Analysis 2 excluded patients with prior neurosurgical resection or WBRT. All risk factors were entered into univariate analysis using the Cox proportional hazards model. Significant risk factors were further entered into multivariate analysis, except GPA and RPA scores given these measures represent a composite of other investigated factors. JMP Pro 13 (SAS Institute Cary, NC) was used to perform the analyses. All *P*‐values were two‐sided with a significance level of 0.05.

## Results

Of 1017 eligible patients treated with SRS for BM between 1994 and 2015, patients with one lesion (543 patients) and patients missing survival or baseline data (83 patients) were excluded, thereby resulting in 391 patients who were included in the analysis.

All patients were analyzed regardless of prior WBRT or neurosurgical resection. Baseline characteristics are presented in Table 1. Patients with 5 or more BM had a higher rate of untreated BM and progressive BM from baseline as well as a lower GPA compared to patients with 2–4 BM. Of note, there were no significant differences in total tumor volume between the cohorts.

**Table 1:** Baseline patient and treatment characteristics

	2–4 (*n* = 314)	5+ (*n* = 77)	All (*n* = 391)	*P*‐value
Age, median (range)	60 (13–100)	55 (29–100)	59 (13–100)	0.11
Sex				
Male	172 (55%)	45 (58%)	217 (56%)	0.56
Female	142 (45%)	32 (42%)	174 (44%)	
Histology				
Breast adenocarcinoma	46 (15%)	9 (12%)	55 (14%)	0.26
Lung NSCLC	59 (19%)	10 (13%)	69 (18%)	
Melanoma	140 (45%)	45 (60%)	185 (48%)	
Renal cell carcinoma	25 (8%)	4 (5%)	29 (8%)	
Other	39 (13%)	7 (9%)	46 (12%)	
KPS median (range)	90 (50–100)	90 (40–100)	90 (40–100)	0.89
GPA, median (range)	2 (0–3.5)	1.5 (0.5–3)	1.5 (0–3.5)	<0.0001
RPA				
Class 1	46 (15%)	6 (8%)	52 (14%)	0.20
Class 2	247 (80%)	66 (89%)	313 (82%)	
Class 3	15 (5%)	2 (3%)	17 (4%)	
Brain mets diagnosed within 3 months of primary	42 (16%)	11 (17%)	53 (16%)	0.88
Increase in # of brain mets from baseline to treatment	101 (32%)	47 (61%)	148 (38%)	<0.0001
Untreated brain mets	48 (15%)	19 (25%)	67 (17%)	0.05
Infratentorial brain mets	113 (36%)	33 (43%)	146 (37%)	0.06
SRS dose, Gy, median (range)	18 (12–22)	18 (14–20)	18 (12–22)	0.07
Total tumor volume, cc, median (range)	3.36 (0.07–36.02)	3.53 (0.29–81.60)	3.41 (0.071–81.60)	0.43
Total tumor volume, cc				
<5	195 (62%)	44 (58%)	239 (61%)	0.67
5–10	58 (19%)	14 (18%)	72 (19%)	
>10	60 (19%)	18 (24%)	78 (20%)	

NSCLC, Nonsmall Cell Lung Cancer; KPS, Karnofsky Performance Status; GPA, Grade Prognostic Assessment; RPA, Recursive Partitioning Analysis; mets, metastases; SRS, Stereotactic Radiosurgery.

**Figure 1:**
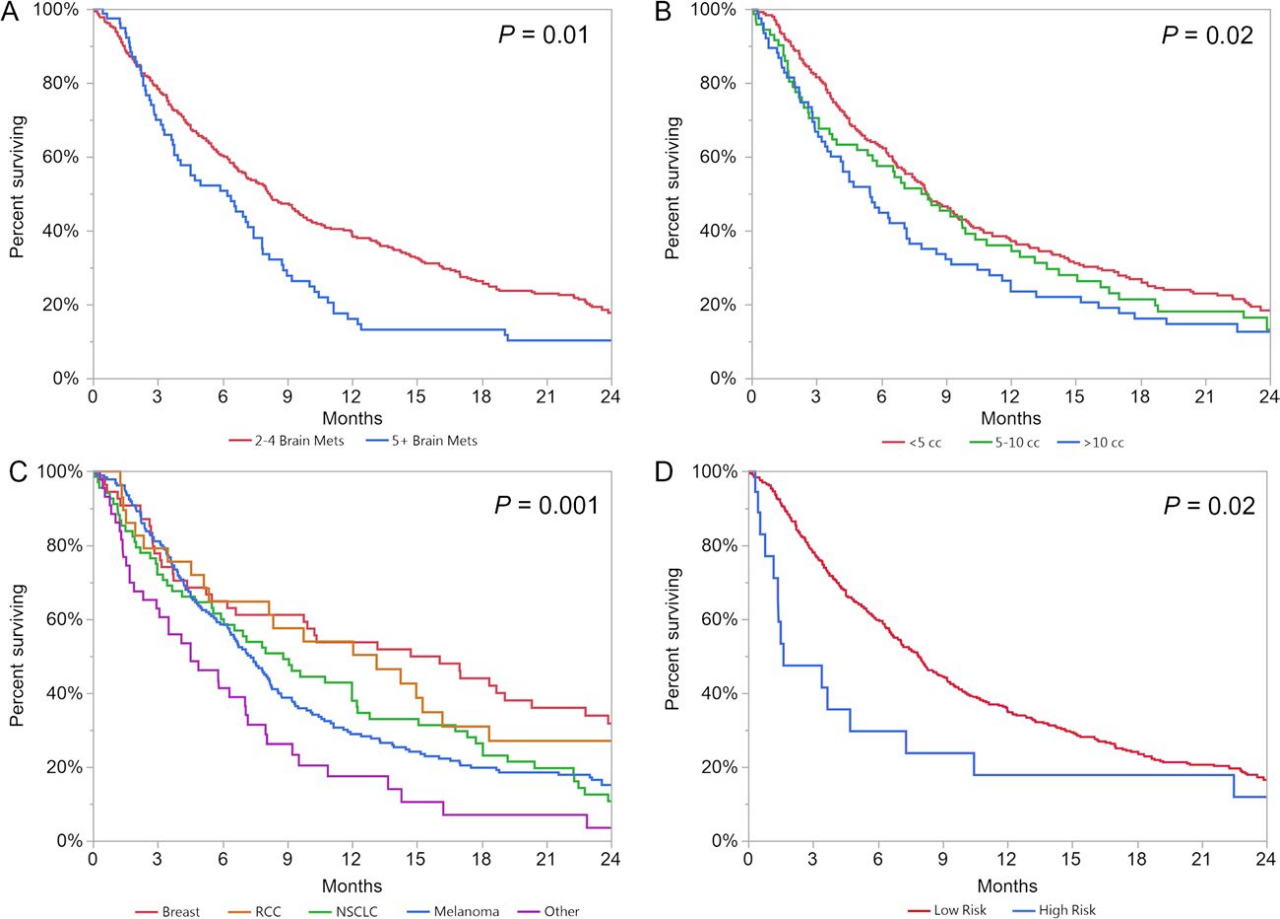
Kaplan–Meier survival curves of (A) number of brain metastases by category (B) tumor volume by category (C) histology (D) risk category.

With a median follow‐up of 7.1 months, patients with 2–4 BM (*n* = 314) had a median OS of 8.1 months and patients with 5 or more BM (*n* = 77) had a median OS of 6.2 months (*P* = 0.0136) (Fig. 1A). Prognostic factors with a significant effect on OS on univariate analysis included lesion number, total tumor volume, histology, untreated BM, KPS, GPA, and RPA. Tumor volume >10 cc had a hazard ratio (HR) of 1.451 for worse OS when compared to tumor volume <5 cc (*P* = 0.010) (Fig. 1B). Tumor volume was also significantly associated with worse OS when analyzed as a continuous variable (*P* = 0.0314). Breast histology had the best OS with melanoma, lung, and other categories faring significantly worse (Fig. 1C). Lung primaries (nonsmall cell lung cancer) and other histologies (including small cell lung cancer, colorectal, prostate, ovarian) had the worst outcomes with HRs of 1.557 and 2.463, respectively, in comparison with patients with breast cancer. On multivariate analysis, only total tumor volume, KPS, and histology remained significant (Table 2).

**Table 2:** Univariate and multivariate Cox proportional hazards model of overall survival

Variables	Univariate		Multivariate	
	HR (95% CI)	*P*‐value	HR (95% CI)	*P*‐value
Number of BM treated				
2–4	Ref.	Ref.	Ref.	Ref.
5+	1.396 (1.062–1.810)	0.0176	1.220 (0.903–1.624)	0.1921
Age				
≤60 years	Ref.	Ref.	Ref.	Ref.
>60 years	1.228 (0.991–1.521)	0.0604	1.201 (0.957–1.505)	0.1143
Sex				
Male	Ref.	Ref.	–	–
Female	0.877	0.2272	–	–
Histology				
Breast adenocarcinoma	Ref.	Ref.	Ref.	Ref.
Lung NSCLC	1.557 (1.059–2.291)	0.0236	1.664 (1.110–2.497)	0.0134
Melanoma	1.473 (1.059–2.049)	0.0173	1.422 (0.997–2.009)	0.0397
Renal cell carcinoma	1.180 (0.725–1.922)	0.5085	1.053 (0.641–1.730)	0.8395
Other	2.463 (1.596–3.800)	<0.0001	1.947 (1.231–3.079)	0.0049
KPS				
90–100	Ref.	Ref.	Ref.	Ref.
70–80	1.533 (1.219–1.922)	0.0003	1.417 (1.111–1.802)	0.0052
≤60	2.690 (1.542–4.369)	0.0010	2.340 (1.301–3.912)	0.0059
GPA				
0–1	Ref.	Ref.	–	–
1.5–2.5	0.686 (0.537–0.883)	0.0037	–	–
3–4	0.514 (0.330–0.777)	0.0013	–	–
RPA				
Class 1	Ref.	Ref.	–	–
Class 2	1.129 (0.827–1.579)	0.4546	–	–
Class 3	2.394 (1.305–4.177)	0.0059	–	–
Synchronous BM	1.139 (0.815–1.556)	0.4351	–	–
Increase in number of BM from baseline to treatment	0.908 (0.725–1.131)	0.3911	–	–
Untreated BM	1.436 (1.078–1.882)	0.0142	1.321 (0.977–1.757)	0.0701
Infratentorial BM	1.140 (0.915–1.415)	0.2418	–	–
SRS dose	0.961 (0.915–1.013)	0.1394	–	–
SRS dose				
<18 Gy	Ref.	Ref.	–	–
≥18 Gy	1.141 (0.870–1.477)	0.3323	–	–
Total tumor volume				
<5 ccs	Ref.	Ref.	Ref.	Ref.
5–10 ccs	1.223 (0.916–1.610)	0.1682	1.326 (0.979–1.768)	0.0678
>10 ccs	1.451 (1.094–1.900)	0.0103	1.641 (1.217–2.186)	0.0014

NSCLC, Nonsmall Cell Lung Cancer; KPS, Karnofsky Performance Status; GPA, Grade Prognostic Assessment; RPA, Recursive Partitioning Analysis; mets, metastases; SRS, Stereotactic Radiosurgery.

**Figure 2:**
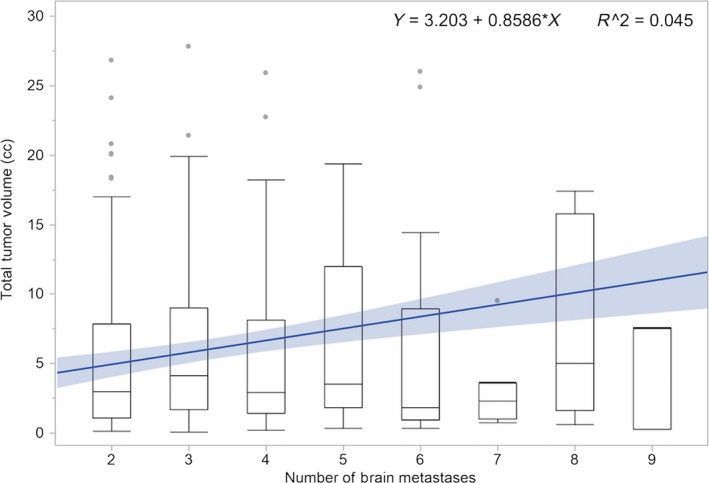
Box plot of tumor volume versus number of brain metastases with linear regression.

To further analyze the relationship of tumor volume and lesion number, a standard least squared regression model was used. There was a statistically significant but weak correlation between the two (*R* ^2^ = 0.045, *P* < 0.0001). The number of BM only accounted for 4.5% of the variance in total tumor volume. Figure 2 displays this relationship of volume by lesion number in a box plot.

After excluding 121 patients with prior surgery (*n* = 77) and/or WBRT (*n* = 58), the difference in median OS was not significantly different on univariate (*P* = 0.0603) or multivariate (*P* = 0.2772) analysis when comparing 2–4 BM (*n* = 219) to 5 or more BM (*n* = 52) (Tables S1 and S2). Histology, KPS, GPA, RPA, and total tumor volume were significantly associated with OS on univariate analysis. On multivariate analysis, total tumor volume (both as a categorical and as a continuous variable), KPS, and histology remained significant, consistent with the primary analysis of all patients.

We further analyzed the data to attempt to develop a metric for identifying patients with the worst prognosis where consideration of best supportive care may be warranted. Given the median OS of breast cancer histology (14.72 months) versus all nonbreast histology (median OS 7.18 months; *P* = 0.006), we examined all patients with nonbreast cancer. Seventy‐eight patients with nonbreast histology had OS <3 months. Of these, 21 had total tumor volume >10 cc. Nine had total tumor volume >10 cc and KPS <80. We found those with total tumor volume >10 cc and KPS <80 had a median OS of 1.63 months compared to 7.87 months for all patients that did not meet all these criteria (*P* = 0.02). The KM survival curves based on these criteria are presented in Figure 1D.

## Discussion

Many radiation oncologists use lesion number as an important consideration when deciding on an optimal treatment strategy of SRS versus WBRT for BM. In a survey of practicing radiation oncologists by Sandler et al., number of lesions was identified as the *most important* factor in decision making for selecting WBRT versus SRS—more important than performance status, size of lesions, extracranial disease status, histology, and patient convenience. Furthermore, non‐CNS specialists (as defined by patient volume) were more likely to pick a lower number for the cutoff of when to no longer treat with SRS (mean 5.1 BM) versus CNS specialists (mean 8.1 BM), with a mean cutoff closer to four for minimal volume providers. Most radiation oncologists listed four to six lesions as the cohort they found most challenging in deciding how to treat 4. However, prior studies as well as our analysis demonstrate that although lesion number is an easily quantifiable metric, it is not necessarily the best factor in determining optimal candidacy for SRS.

In a prospective observational trial (JLGK0901), patients with 2–4 BM (*n* = 531) and 5 or more BM (*n* = 208) had equivalent median survivals of 10.8 months (*P* = 0.78). There was no difference in the rate of neurologic death or local recurrence between the two groups 10. Our findings are consistent with these results. Although median OS for all patients significantly favored the 2–4 group on univariate analysis (8.13 months vs. 6.23 months, *P* = 0.0176), on MVA, the difference was no longer statistically significant (*P* = 0.2714). This result was true for all patients including those with prior WBRT and/or surgery as well as when patients were excluded that had prior WBRT and/or surgery. For patients not receiving prior WBRT and/or surgery, lesion number was not significant on univariate (*P* = 0.0603) or MVA (*P* = 0.2772).

Yamamoto et al. concluded that radiosurgery alone for patients with 5 or more BM was noninferior to outcomes with SRS alone in patients with 2–4 BM. Their group further evaluated patients with *10 or more* BM treated with SRS in a propensity score case‐matched analysis and found no difference between groups for OS or neurologic death as well as other measures such as local recurrence, repeat SRS for new lesions, or complications 9. Additional evidence comes from prior retrospective series including Chang et al. 14, who found no difference in regard to outcomes after SRS for patients with 1–5, 6–10, 11–15 or even more metastases.

Tumor volume may be more important in terms of prognostication than lesion number. In the current series, tumor volume >10 cc was associated with worse OS (HR = 1.451). Multiple prior series have found similar results regarding the association between increasing tumor volume and worse outcomes. Bhatnagar et al., Likhacheva et al., and Baschnagel et al., demonstrated that tumor volume was statistically significantly associated with OS while number of BM was not 15, 16, 17. Interestingly, this was true in our series of all patients and excluding patients with prior WBRT or surgery. Cumulative tumor volume was likewise significantly associated with worse survival on univariate analysis in JLGK0901.

Secondary factors including female sex, age <65, KPS ≥80, stable extracranial disease, and the absence of neurologic symptoms were significantly associated with longer OS in JLGK0901 10. In our analysis, histology and KPS (along with tumor volume) were significantly associated with OS on MVA. These factors have been studied in prior series and components utilized in the graded prognostic assessment and in nomograms. While lesion number is often a factor, tumor volume is rarely included in these scoring systems 18.

To further investigate the association of the interplay of number of BM and volume, we looked at the correlation between the two. While increasing number of BM was significantly correlated with tumor volume, it accounted for only 5% of the variance. Additionally, we identified a high‐risk cohort, namely histology other than breast cancer, tumor volume >10 cc, and KPS <80. These patients had a median OS of <2 months.

This study is limited by its retrospective nature and the limited scope of patient data, such as lack of information regarding systemic therapies. These data are subject to inherent biases including selection bias in that the 5 or more BM group could represent a select and more favorable cohort. However, the intent of this analysis was to look at patients in a clinical setting treated at the discretion of the consulting radiation oncologist, and the groups were well balanced with the exception of GPA as described above. Patients treated over the course of two decades were analyzed, representing a heterogeneous group with many changes in overall approaches to oncologic care throughout this time period. However, these results of patients treated at a single institution over 20 years have practical implications for current management of the increasingly common patient presenting with multiple BM, especially in the absence of a randomized trial. Arbitrarily using a cutoff of 5 or more metastases is not warranted based on this data and should not exclude patients from radiosurgery, nor should prior WBRT or neurosurgical resection.

Current guidelines have clearly delineated recommendations for patients with 4 or fewer metastases but not for patients with 5 or more lesions in regard to SRS 19, 20. A single institution phase III randomized trial (NCT01592968) currently enrolling patients with 4 or more BM to SRS versus WBRT will ideally provide level 1 evidence regarding optimal management 21.

Ultimately, the decision to treat a patient with more than 4 BM with SRS depends on a number of clinical and pathologic factors, including systemic therapeutic options, prognosis, and the patient's goals of care. These factors should be discussed in a multidisciplinary tumor board setting when possible. Tumor volume in conjunction with other clinical and pathologic features is likely more important in terms of prognostication than lesion number alone, and has recently been shown to improve prognostic models including disease‐specific GPA 22, 23, 24.

With the increasingly common use of targeted agents, delays in systemic therapy secondary to WBRT could ultimately be detrimental to a patient's outcome. Nevertheless, even in the setting of targeted agents, radiotherapy remains an important treatment for patients with BM and could impact OS 25. Furthermore, immunotherapy and targeted agents may lead to better tumor control and reduce distant brain failure, rendering SRS more impactful 26. Finally, with close surveillance, patients that have regional failure after SRS can undergo effective salvage SRS with low morbidity, providing further rationale for the upfront use of SRS.

## Conclusions

Patients with 5 or more BM treated with SRS have comparable OS to those with 2–4 BM, regardless of prior WBRT or surgery, and remain good candidates for SRS based on the results of this study. While number of lesions may be prognostic, total tumor volume may be a more important factor in determining OS. Given the side effects associated with WBRT and equivalent outcomes with SRS alone, our analysis supports offering SRS alone to select patients with 5 or more BM with total tumor volume <10 cc in clinical practice.

## Conflict of Interest

Eric L. Chang discloses that he received speaker's honorarium from Brainlab.

## Supporting information

**Table S1.** Baseline patient and treatment characteristics, after excluding patients receiving prior surgery or WBRT.
**Table S2.** Univariate and multivariate Cox proportional hazards model of overall survival, after excluding patients receiving prior surgery or WBRT.
