# Supplementary figures and images for: The growing importance of lesion volume as a prognostic factor in patients with multiple brain metastases treated with stereotactic radiosurgery
Source: Cancer Med. 2018 Feb 14;7(3):757–64. doi: 10.1002/cam4.1352 (PMC5852368; doi:10.1002/cam4.1352)

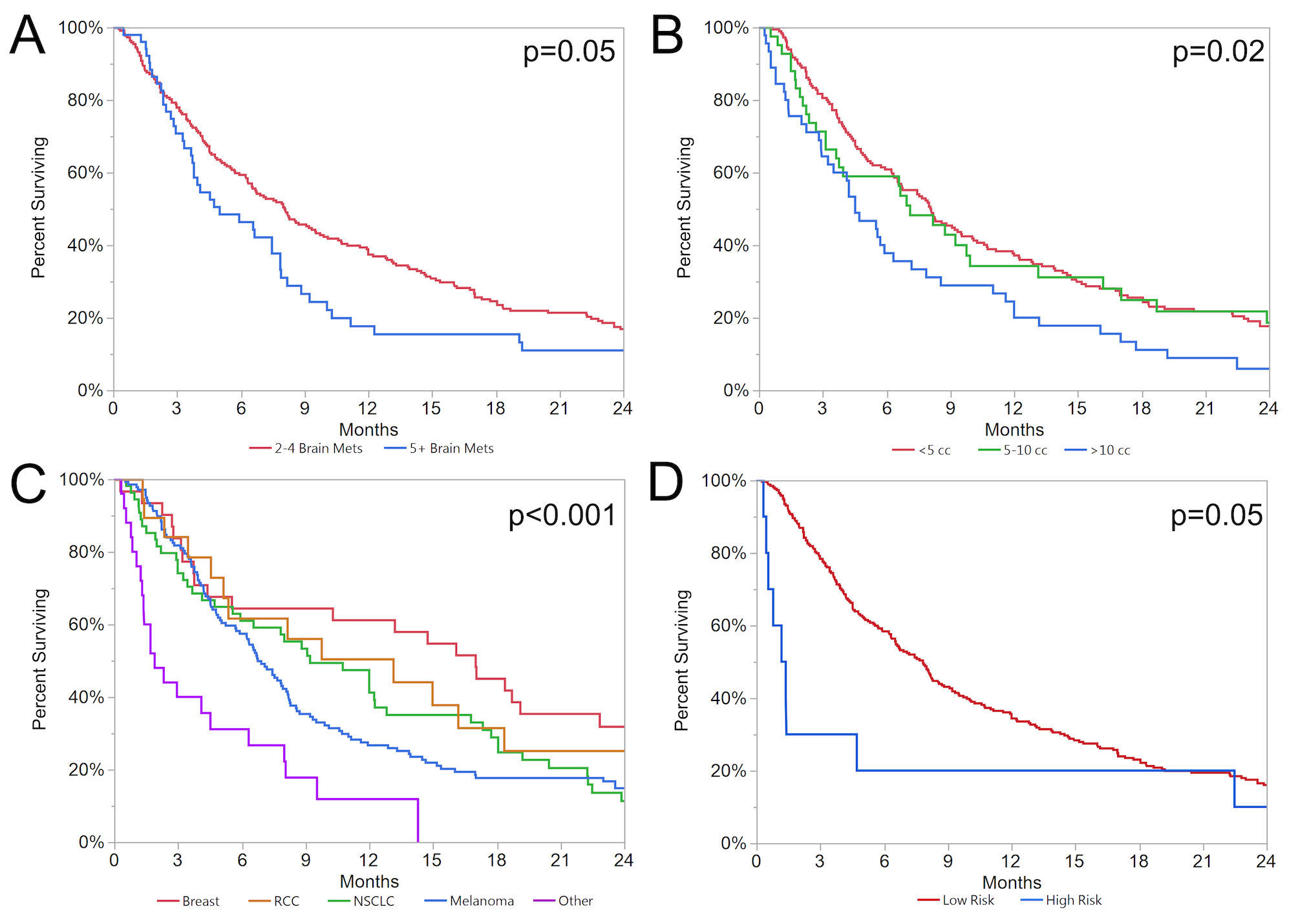

Supplement: Supplementary file 1 [file CAM4-7-757-s001.tiff]

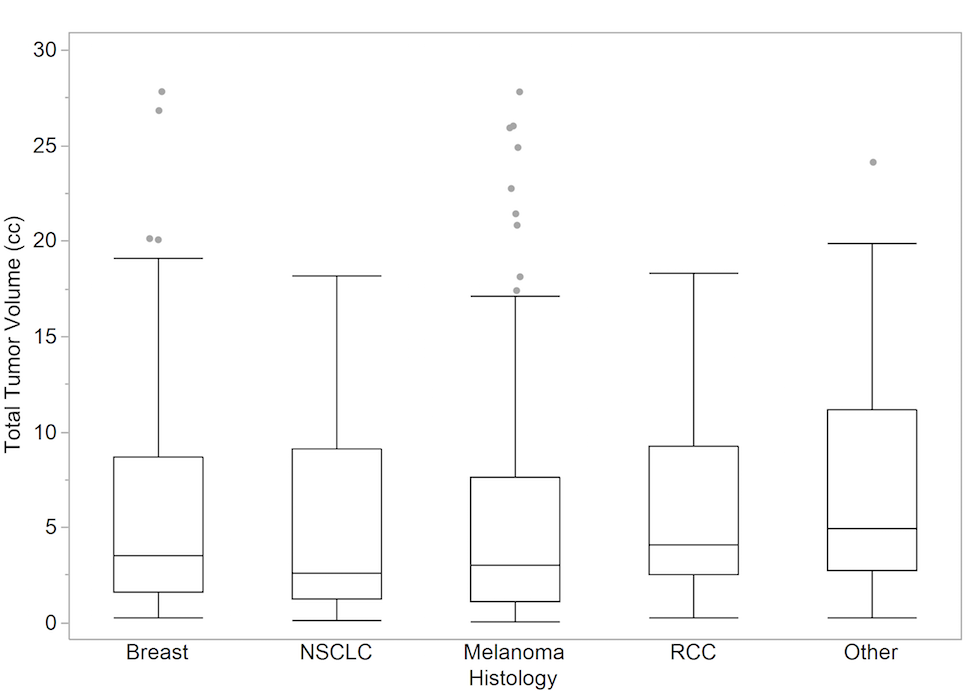

Supplement: Supplementary file 2 [file CAM4-7-757-s002.tiff]
