# Supplementary material for: The growing importance of lesion volume as a prognostic factor in patients with multiple brain metastases treated with stereotactic radiosurgery
Source: Cancer Med. 2018 Feb 14;7(3):757–64. doi: 10.1002/cam4.1352 (PMC5852368; doi:10.1002/cam4.1352)
Supplement: Supplementary file 3 — Table S1. Baseline patient and treatment characteristics, after excluding patients receiving prior surgery or WBRT. Table S2. Univariate and multivariate Cox proportional hazards model of overall survival, after excluding patients receiving prior surgery or WBRT. [file CAM4-7-757-s003.docx]

**Supplemental Table 1. Baseline Patient and Treatment Characteristics, After Excluding Patients Receiving Prior Surgery or WBRT**

|  | **2-4 (n=219)** | **5+ (n=52)** | **All (n=271)** | ***P*-value** |
| --- | --- | --- | --- | --- |
| Age, median (range) | 61 (13-100) | 56.5 (29-86) | 60 (13-100) | 0.08 |
| Sex |  |  |  | 0.78 |
| Male | 131 (60%) | 30 (58%) | 161 (59%) |  |
| Female | 188 (40%) | 22 (42%) | 110 (41%) |  |
| Histology |  |  |  | 0.63 |
| Breast adenocarcinoma | 25 (12%) | 6 (12%) | 31 (12%) |  |
| Lung NSCLC | 47 (22%) | 8 (16%) | 55 (21%) |  |
| Melanoma | 106 (49%) | 31 (61%) | 137 (51%) |  |
| Renal cell carcinoma | 16 (7%) | 3 (6%) | 19 (7%) |  |
| Other | 23 (11%) | 3 (6%) | 26 (10%) |  |
| KPS median (range) | 90 (50-100) | 90 (40-100) | 90 (40-100) | 0.74 |
| GPA, median (range) (IQR) | 1.5 (0-3.5) (3-5) | 1.5 (0.5-3) (2-4) | 1.5 (0-3.5) | **0.0001** |
| RPA |  |  |  | 0.85 |
| Class 1 | 28 (13%) | 5 (10%) | 33 (13%) |  |
| Class 2 | 177 (83%) | 42 (86%) | 219 (83%) |  |
| Class 3 | 9 (4%) | 2 (4%) | 11 (4%) |  |
| Brain mets diagnosed within 3 months of primary | 31 (17%) | 6 (14%) | 37 (16%) | 0.65 |
| Increase in # of brain mets from baseline to treatment | 67 (31%) | 32 (62%) | 99 (37%) | **<0.0001** |
| Untreated brain mets | 23 (11%) | 12 (23%) | 35 (13%) | **0.02** |
| Infratentorial brain mets | 72 (33%) | 21 (40%) | 93 (34%) | 0.31 |
| SRS dose, Gy, median (range) (IQR) | 18 (12-22) (18-20) | 18 (14-20) (18-18) | 18 (12-22) | **0.01** |
| Total tumor volume, cc, median (range) | 2.84 (0.07-36.02) | 3.11 (0.29-81.60) | 2.84 (0.071-81.60) | 0.55 |

Abbreviations: NSCLC, Non-Small Cell Lung Cancer; KPS, Karnofsky Performance Status; GPA, Grade Prognostic Assessment; RPA, Recursive Partitioning Analysis; mets, metastases; SRS, Stereotactic Radiosurgery

**Supplemental Table 2. Univariate and Multivariate Cox Proportional Hazards Model of Overall Survival, After Excluding Patients Receiving Prior Surgery or WBRT**

|  | **Univariate** | | **Multivariate** | |
| --- | --- | --- | --- | --- |
| **Variables** | **HR (95% CI)** | ***P*-value** | **HR (95% CI)** | ***P*-value** |
| Number of BM treated |  |  |  |  |
| 2-4 | Ref. | Ref. | Ref. | Ref. |
| 5+ | 1.379 (0.986-1.89) | 0.0603 | 1.226 (0.844-1.740) | 0.2772 |
| Age |  |  |  |  |
| ≤60 years | Ref. | Ref. | Ref. | Ref. |
| >60 years | 1.169 (0.904-1.510) | 0.2338 | 1.079 (0.816-1.423) | 0.5945 |
| Sex |  |  |  |  |
| Male | Ref. | Ref. | - | - |
| Female | 0.845 (0.650-1.094) | 0.2023 | - | - |
| Histology |  |  |  |  |
| Breast adenocarcinoma | Ref. | Ref. | Ref. | Ref. |
| Lung NSCLC | 1.568 (0.969-2.538) | 0.0622 | 1.681 (1.013-2.790) | **0.0413** |
| Melanoma | 1.670 (1.089-2.561) | **0.0134** | 1.611 (1.032-2.513) | **0.0282** |
| Renal cell carcinoma | 1.301 (0.697-2.430) | 0.4138 | 1.284 (0.668-2.468) | 0.4577 |
| Other | 4.194 (2.337-7.525) | **<0.0001** | 3.290 (1.731-6.254) | **0.0004** |
| KPS |  |  |  |  |
| 90-100 | Ref. | Ref. | Ref. | Ref. |
| 70-80 | 1.495 (1.124-1.974) | **0.0061** | 1.404 (1.029-1.900) | **0.0326** |
| ≤60 | 2.418 (1.227-4.283) | **0.0129** | 2.067 (1.005-3.878) | **0.0486** |
| GPA |  |  |  |  |
| 0-1 | Ref. | Ref. | - | - |
| 1.5-2.5 | 0.710 (0.531-0.961) | **0.0272** | - | - |
| 3-4 | 0.540 (0.310-0.892) | **0.0153** | - | - |
| RPA |  |  |  |  |
| Class 1 | Ref. | Ref. | - | - |
| Class 2 | 1.080 (0.739-1.641) | 0.7023 | - | - |
| Class 3 | 2.389 (1.100-4.775) | **0.0291** | - | - |
| Synchronous BM | 0.961 (0.660-1.447) | 0.8415 | - | - |
| Increase in number of BM from baseline to treatment | 1.056 (0.806-1.375) | 0.6884 | - | - |
| Untreated BM | 1.372 (0.932-1.956) | 0.1058 | 1.138 (0.745-1.689) | 0.5386 |
| Infratentorial BM | 1.103 (0.843-1.434) | 0.4721 | - | - |
| SRS dose |  |  |  |  |
| <18 Gy | Ref. | Ref. | - | - |
| ≥18 Gy | 1.010 (0.695-1.425) | 0.9579 | - | - |
| Total tumor volume |  |  |  |  |

**Supplemental Table 2 Continued**

|  | **Univariate** | | **Multivariate** | |
| --- | --- | --- | --- | --- |
| **Variables** | **HR (95% CI)** | ***P*-value** | **HR (95% CI)** | ***P*-value** |
| Total tumor volume |  |  |  |  |
| <5 ccs | Ref. | Ref. | Ref. | Ref. |
| 5-10 ccs | 1.120 (0.764-1.597) | 0.5497 | 1.244 (0.830-1.808) | 0.2817 |
| >10 ccs | 1.631 (1.148-2.269) | **0.0071** | 1.987 (1.360-2.844) | **0.0005** |

Abbreviations: NSCLC, Non-Small Cell Lung Cancer; KPS, Karnofsky Performance Status; GPA, Grade Prognostic Assessment; RPA, Recursive Partitioning Analysis; mets, metastases; SRS, Stereotactic Radiosurgery
